# Outdoor Endurance Training with Air Pollutant Exposure Versus Sedentary Lifestyle: A Comparison of Airway Immune Responses

**DOI:** 10.3390/ijerph16224418

**Published:** 2019-11-12

**Authors:** Juliana de Melo Batista dos Santos, Roberta Foster, Anne-Charlotte Jonckheere, Marcelo Rossi, Luiz Antonio Luna Junior, Catherine Machado Katekaru, Matheus Cavalcante de Sá, Lucas Guimarães Pagani, Francine Maria de Almeida, Jônatas do Bussador Amaral, Rodolfo de Paula Vieira, Andre Luis Lacerda Bachi, Dominique Magdalena A Bullens, Mauro Vaisberg

**Affiliations:** 1 ENT Lab, Department of Otorhinolaryngology, Federal University of São Paulo (UNIFESP), Rua dos Otonis, 700, Piso superior/Second floor, Sao Paulo SP CEP 04025-002, Brazil; 2 Method Faculty of Sao Paulo (FAMESP), Av. Jabaquara, 1314, Sao Paulo SP 04046-200, Brazil; 3 Allergy and Clinical Immunology Research Group, Department of Microbiology, Immunology and Transplantation, KU Leuven, UZ Herestraat 49 box 811, 3000 Leuven, Belgium; 4 Medicine School, São Paulo University, Av. Dr. Arnaldo, 455—Cerqueira César, São Paulo SP CEP 01246-903, Brazil; 5 Post-graduation Program in Science of Human and Rehabilitation, Federal University of São Paulo (UNIFESP), Av. Ana Costa, 95—Vila Mathias, Santos SP CEP 11060-001, Brazil; 6 Brazilian Institute of Teaching and Research in Pulmonary and Exercise Immunology (IBEPIPE), Rua Pedro Ernesto 240, São José dos Campos SP CEP 12245-520, Brazil; 7 Post-graduation Program in Bioengineering and Biomedical Engineering, Universidade Brasil, Rua Carolina Fonseca, 584—Itaquera, São Paulo SP CEP 08230-030, Brazil; 8 School of Medicine, Anhembi Morumbi University, R. Jaceru, 247, São José dos Campos SP CEP 04705-000, Brazil; 9 Clinical Division of Pediatrics, UZ Leuven, Herestraat 49, 3000 Leuven, Belgium

**Keywords:** runners, air pollutants, cytokines, antibacterial peptides, fractional exhaled nitric oxide, Th immune response

## Abstract

Although regular exercise-training improves immune/inflammatory status, the influence of air pollutants exposure during outdoor endurance training compared to a sedentary lifestyle has not yet been clarified. This study aimed to compare the immune/inflammatory responses in the airways of street runners and sedentary people after acute and chronic particulate matter (PM) exposure. Forty volunteers (street runners (RUN, n = 20); sedentary people (SED, n = 20)) were evaluated 1 (acute) and 10 (chronic) weeks after PM exposure. Cytokines [interferon (IFN)-γ, tumor necrosis factor (TNF)-α, interleukin (IL)-6, IL-10, IL-13, and IL-17A] in nasal lavage fluid, salivary antibacterial peptides (lactoferrin (LTF), cathelicidin (LL-37), defensin-α 1–3), and secretory immunoglobulin A (SIgA), plasma club cell protein (CC16), and fractional exhaled nitric oxide (FeNO) were analyzed. After acute exposure, the RUN group showed lower levels of IL-13, IL-10, and FeNO, but higher defensin-α than the SED group. After chronic exposure, the RUN group showed elevation of IFN-γ, IL-10, IL-17A, and a decrease of FeNO levels, whereas the SED group showed elevation of TNF-α, IL-6, IL-10, and a decrease of IL-13 levels. Comparing these groups, the RUN group showed higher levels of SIgA and LTF, and lower FeNO levels than the SED group. In relation to the Th immune response analysis after acute and chronic PM exposure, the RUN group showed a pattern associated with Th1, while in the SED group, a Th2 pattern was found. Both groups showed also a Th17 immune response pattern. Our results allow us to suggest that the immune/inflammatory status of the respiratory tract after acute and chronic PM exposure was improved by the long-standing regular practice of outdoor endurance exercise compared to a sedentary lifestyle.

## 1. Introduction

It is widely accepted that air pollution represents the biggest environmental risk to health, mainly associated with the development of respiratory tract illness [1]. In accordance with the World Health Organization (WHO) [2], the increasing number of acute air pollution episodes in many cities around the world has been worrying, which leads to a significant excess of mortality or morbidity. Among several pollutants, including nitrogen oxide, ozone, carbon monoxide, and sulfur dioxides, the most frequently used as an indicator of exposure to air pollution, in general, is the particulate matter (PM) [2,3]. Furthermore, it has been demonstrated that exposure to fine PM (PM_2.5_) accounts for the sixth-highest mortality risk in 2016 and contributed to 4.1 million deaths, accounting for 7.5% of total global deaths [4].

Particulate matters are easily deposited in bifurcations or branches of the bronchial tree, causing local damage due to their interaction with the epithelial surface and mucosa of the bronchi [5,6]. The deleterious effect of particulate pollution has been reported to be associated with its particle size and/or chemical composition, as well as the physical properties of the particles (including mass, volume, surface area, and the number of particles), which may influence their retention in the lung [7]. In addition, PM can alter the permeability of epithelial cells throughout a redox imbalance [8], as well as promoting an inflammatory response in animals and humans [2,9,10]. Indeed, PM can induce the transcription factor activation of proinflammatory genes that lead to the increase of fractional exhaled nitric oxide (FeNO), C-reactive protein (CRP), fibrinogen, and proinflammatory cytokines [interferon gamma (INF-γ), tumor necrosis factor-alpha (TNF-α), interleukin-1 (IL-1), and IL-6 [7,11,12,13].

From the 1980s [14,15], it has been shown that air pollutant exposure during exercise can decrease the pulmonary and vascular function in healthy individuals. It appears to be related to an increased systemic, and in airways, oxidative stress and inflammation, compromising sports performance [16,17]. Based on the fact that environmental factors, especially air pollution, can affect the health and sports performance of athletes, the announcement that the Olympic Games would be held in the city of Beijing reached high attention by all countries [18,19,20].

This concern led the Chinese government to develop a program named “Air Quality Guarantee Plan for the 29th Olympics in Beijing” to minimize air pollutant emissions in both Beijing and surrounding areas before and during Olympic and Paralympic Games [21]. The consequent decrease of air pollutant levels was associated with a reduction in the acute effects of air pollutants in several biomarkers of pulmonary and systemic inflammation in healthy young adults [17].

In fact, during exercise, increased ventilation occurs that may result in a greater influx of air and pollutants into the airways, and those might even reach systemic circulation [22,23,24]. So, performing outdoor endurance exercise can lead to an increase in the total number of particles deposited in the respiratory tract that may exceed 4.5 times than observed at rest [24]. Beyond the high rate of ventilation, a change from nasal to oral breathing associated with an increase of airflow speed, carry the pollutants deeper into the lungs [25], further amplifying the dose of pollutants during endurance physical activity.

Based on the negative effect of air pollutant exposure in health, it generally is advised to avoid practicing exercise in polluted areas [26]. Unfortunately, some areas in the world with numerous inhabitants are polluted for a longer period of the year. Nevertheless, there is no consensus on the beneficial or harmful effects of exercise performed in such a polluted environment, by comparing the health-related aspects of air pollution exposure in athletes and sedentary individuals living in those polluted environments.

Roberts et al. [27] analyzed the association between air pollution and physical inactivity in adults in the United States, and concluded that increased levels of PM_2.5_, PM_10,_ and O_3_ are associated with reduced physical activity. It is worth mentioning that physical inactivity accounts for 9% of premature deaths, or more than 3.5 million of the 57 million deaths worldwide in 2008 [28]. The same study estimates that eliminating physical inactivity would increase the life expectancy of the world’s population by 0.68 years and over 1.3 million deaths would be avoided each year.

Tainio et al. [29] investigated the risk of physical activity (walking and biking) for active transport in urban centers and demonstrated that its benefits generally outweigh the health risks caused by air pollution and therefore should be encouraged. Moreover, Vieira et al. [12] demonstrated that aerobic exercise inhibits pulmonary inflammation, the release of pro-inflammatory pulmonary and systemic cytokines, as well as pulmonary oxidative and nitrosative stress levels in a murine model of chronic exposure to diesel exhausted particles.

Taking into account this information, the current study aimed to evaluate the immune/inflammatory airway response in street runners and sedentary people after acute and chronic periods of particulate matter exposure. So, our hypothesis is that even though the practice of outdoor aerobic exercises is associated with increased inhalation of air pollutants due to hyperventilation, physical training would be able to mitigate the deleterious effects of air pollutant exposure by modulating the airways immune/inflammatory responses differently from that found in sedentary people.

## 2. Material and Methods

**Figure 1:**
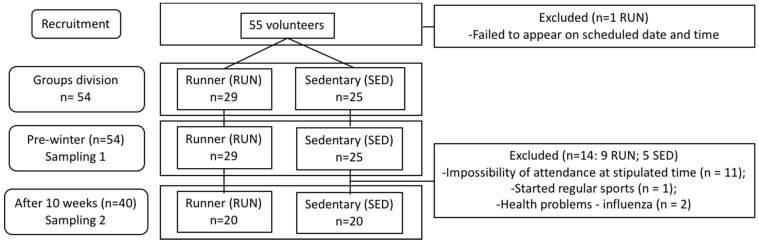
Flow diagram of the study.

As represented in the flow diagram (Figure 1), forty volunteers, both male and female (aged between 18 and 55 years), residents of the Metropolitan Area of São Paulo (MASP), Brazil were enrolled in this study. All volunteers were recruited through electronic media using the Press Office of the Federal University of São Paulo and also through sports advisers and athletes registered at Corpore, a non-profit entity. To the runners’ group (RUN, n = 20, aged 37.4 ± 8.9) our inclusion criteria were: regularly performed outdoor running, at least for the last 6 months, ≥ 3 times a week and also completed an official 10 km outdoor race in the last 6 months with a minimum average speed of 9 km/h. The selection of volunteers to sedentary group (SED, n = 20, aged 31.8 ± 10.2) following the criteria: not practicing regular physical exercise, at least for the last 12 months. All volunteers signed the Consent Form previously approved by the Ethics Committee of Federal University of São Paulo [CEP-UNIFESP (nº 804.597/2014)], which was in agreement with the Ethical Standards defined by Harris and Atkinson (2015) [30]. Moreover, all experiments were performed in accordance with the Declaration of Helsinki [31].

Volunteers who presented with diagnosis of chronic inflammatory diseases; chronic respiratory and/or heart diseases; or reported chronic use of corticosteroids, anabolic agents, or hormones with action on muscle tissue; current smokers; presence of physical condition that did not permit to carry out the proposed tests; fertile women who did not use hormonal contraceptives and individuals who live or work in regions without a station to monitor air pollutants (considering the network of the Environmental Company of Sao Paulo State (CETESB)) were excluded.

### 2.1. Experimental Design

**Figure 2:**
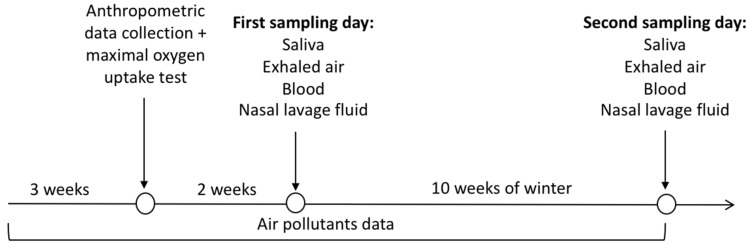
Experimental design.

The first sample collection (pre-winter) of nasal lavage fluid, blood, saliva, and exhaled air was performed two weeks after a maximal cardiopulmonary test and anthropometric data collection and also ten weeks after first sample collection (ten weeks after winter starts) (Figure 2).

The data of the pollutants came from the database of the Environmental Company of São Paulo State (CETESB). Data of 2.5 and 10 particle matter and ozone levels were collected from 20 monitoring stations according to the residence, work, and training sites of all volunteers for 15 weeks (https://servicos.cetesb.sp.gov.br/qa/). The CETESB certified that the monitoring systems strictly followed the quality assurance/quality control (QA/QC) procedure, as approved by the State Council of Environment (CONSEMA) of the State of São Paulo.

Anthropometric characteristics (weight, height, BMI, and total body fat) were evaluated at the beginning of the study to characterize the groups. Total body fat (%) was determined using the BioScan 916 Tetrapolar bioelectrical apparatus (Maltron, United Kingdom), according to the manufacturer’s protocol. To evaluate the maximal oxygen uptake ($\dot{V}$O_2max_), all volunteers performed a cardiopulmonary test on a treadmill (model TR6, Speedo, Nottingham, UK) coupled to a gas analyzer with electrocardiograph monitoring (Oxycon Mobile, Jaeger, Höchberg, Germany; CareFusion Corp, BD Medical Technologies, San Diego, USA), using the Ellestad multi-load protocol [32] following the III Guidelines of the Brazilian Society of Cardiology on ergometric testing [33]. In addition, the “maximum exercise test” was obtained at the moment at which oxygen consumption reached its plateau defined by a respiratory exchange rate (RER) greater than 1.15 L; or when the volunteer reached an RER greater than 1 L together with the predicted maximum heart rate (HR, determined by the Tanaka et al. [34] formula (208 − (0.7 × age)) and validated for the Brazilian population [35]).

### 2.2. Samples Collection

Biological samples (fasting blood, nasal lavage fluid, saliva, and exhaled air) were collected in the beginning and ten weeks after (Figure 2).

Blood samples were collected from a peripheral vein using a tube with an anticoagulating agent (EDTA). Tubes were centrifuged at 2000 rpm at 4 °C for 10 min, and 500 µl of plasma were stored at −80 °C for further analysis of club cell protein (CC16) concentration.

Saliva samples were collected directly into 15 mL Falcon^®^ tubes without stimulation. After that, the samples were centrifuged at 3000 rpm at 4 °C for 10 min, and 500 µl of supernatant was stored at −80 °C for further analysis of secretory immunoglobulin A (SIgA) and antibacterial peptides (defensins-α 1–3, lactoferrin (LTF), and cathelicidin (LL-37)). No buffers or preservatives were added.

Samples of nasal lavage fluid (NLF) were collected by introducing 5 mL of saline solution (0.9% NaCl) into each nostril using a needleless syringe. The volunteers were instructed not to breathe or swallow for 10 s and after this time, return the maximum fluid into a disposable universal collection flask. The collected material was transferred to a graduated polypropylene tube (15 mL Falcon^®^ tube), centrifuged at 3000 rpm at 4 °C for 10 min, and 1000 µl was stored at −80 °C for further analysis of cytokines concentrations. Similarly to saliva, no buffers or preservatives were added [36].

Exhaled air samples used to determine the fractional exhaled nitric oxide (FeNO), were collected following the American Thoracic Society (ATS) and European Respiratory Society (ERS) recommendations [37] and manufacturer’s instructions [38,39].

### 2.3. Determination of Club Cell Protein (CC16)

CC16 concentration was determined in the plasma with an ELISA kit according to the manufacturer’s instructions (BioVendor Research and Diagnostic Products, Brno, Czech Republic).

### 2.4. Determination of Cytokines

Cytokine concentration (IFN-γ, TNF-α, IL-6, IL-10, IL-13, and IL-17A) was determined in the supernatant of NLF using the multiplex U-PLEX MSD (Meso Scale Discovery, Rockville, MD, USA), following the manufacturer’s instructions. The cytokine concentrations obtained initially (pg/mL) in the NLF were normalized by the total protein concentration [cytokine (pg/mL)/ total protein (µg/mL)] using the Bradford method [40].

### 2.5. Determination of Salivary Concentration

The concentration of SIgA and antibacterial peptides (defensins-α 1-3, LTF, and LL-37) were analyzed using commercial ELISA kits (Bioassay Technology Laboratory, Shanghai, China) following the manufacturer’s instructions.

### 2.6. Determination of Fractional Exhaled Nitric Oxide (FeNO)

FeNO levels (recorded in parts per billion, ppb) were determined within four hours after exhaled air collection in the Sievers apparatus [37] by a chemiluminescent test using the NO analyzer (NOA 280; Sievers Instruments Inc., Boulder, CO, USA). During the analysis, a NO analyzer was calibrated using a device that filters the NO present in ambient air and a reference gas with a known concentration of NO (standard mixture for NO calibration at 45 ppb NO2) (White Martins Industrial Gases AS, São Paulo, SP, Brazil). All procedures were in accordance with ATS recommendations for FeNO measurement [37].

### 2.7. Statistical Analysis

Anthropometric data and $\dot{V}$O_2max_ were analyzed using the Student’s t-test. Biological variables from both of the groups (SED and RUN) were analyzed initially by the D’Agostino–Pearson test to assess the normality of the data. Independence among biologicals concentrations and immunological molecule levels were compared by the Welch two-tailed unpaired test, and the strength of associations was assessed by Pearson or Spearman correlation tests, according to the needs. A chi-square test was used to compare proportions. Statistical significance was established at 5.0% level, and all the analysis was performed on the concentration over time data using GraphPad Prism (version 7.0a) software.

## 3. Results

As shown in Table 1, the RUN group showed higher $\dot{V}$O_2max_ than the SED group as expected. In addition, total body fat was higher in the SED group compared to the RUN group. No differences were found in the other physical characteristics between the groups.

### 3.1. Air Pollutants Levels

**Figure 3:**
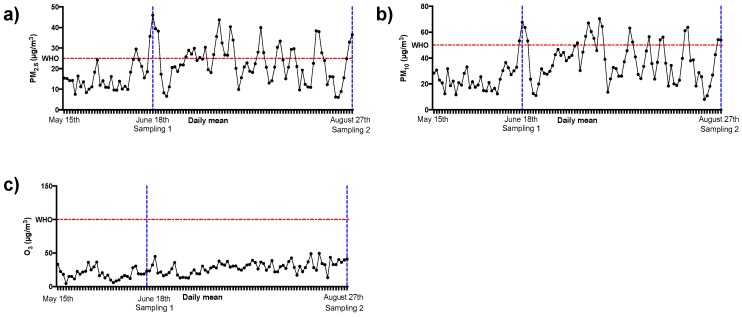
Daily mean levels of each pollutant considered for the study period. The flashing red line refers to the maximum concentration of each pollutant established by WHO [2]. The blue dotted lines refer to the time when the biological samples were collected in the study. (**a**) PM_2.5_ levels; (**b**) PM_10_ levels; (**c**) O_3_ levels.

Figure 3 shows the temporal analysis of pollutant levels during the study period (fifteen weeks).

It is possible to observe that in the week of the first collection there was a peak of particulate matter levels that were higher in comparison to the levels found in the previous four weeks (PM2.5, * *p* = 0.002, and PM10, ^#^ *p* < 0.001) as can be observed in Table 2. During the ten subsequent weeks, a stable period of high particulate matter levels was found (Table 2). Ozone levels remained unchanged during the study and it is worthwhile to highlight that these levels according to WHO were considered “not harmful” [2].

Based on the air pollutant levels present in Table 2, it is clear that the first sampling was performed after acute exposure to air pollutants and the second sampling was performed after chronic exposure to the same pollutants. So, the description of the biological results was named after acute exposure (AE) and after chronic exposure (CE).

Before presenting the results related to the biological parameters studied, it is noteworthy that the statistical analysis between genders showed no significant differences.

### 3.2. Fractional Exhaled Nitric Oxide is Reduced in Runners but not CC16

**Figure 4:**
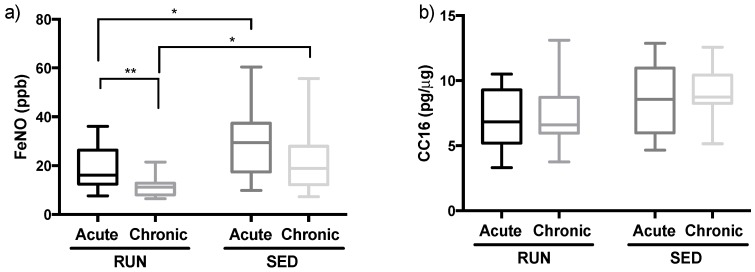
Comparison of fractional exhaled nitric oxide (FeNO) (**a**) and club cell protein (CC16) (**b**) levels after acute and chronic exposure in the RUN and SED groups. All data were analyzed using the Welch two-tailed unpaired test. Values are presented in median and with respective quartile. Level of significance was established at 5% (*p* < 0.05).

As shown in Figure 4a, the RUN group presents lower FeNO levels than SED group both after AE (*p* = 0.04) and CE (*p* = 0.002). In relation to the differences between AE and CE, RUN group showed a significant reduction in the FeNO levels (AE: 18.99 ± 9.07; CE: 11.29 ± 3.96, *p* = 0.003), whereas SED group levels remained unchanged (AE: 28.56 ± 13.61; CE: 21.9 ± 12.7, *p* = 0.137).

According to ATS guidelines [37], FeNO levels above 25 ppb are associated with eosinophilic lung inflammation. In this respect, we observed that the percentage of subjects with FeNO levels above 25 ppb after AE was 35.29% and 50% in RUN and SED groups respectively and no differences between these values were observed (*p* = 0.42). After CE, the number decreased in the RUN group (17.65%), whereas in the SED group the number increased (58.33%) and a significant difference was observed between the groups (*p* = 0.02). No differences were observed between time points in both, RUN and SED groups.

Figure 4b shows that plasma CC16 levels remained unchanged not only between the groups after AE (*p* = 0.06) and after CE (*p* = 0.0889), but also between the time points (RUN, *p* = 0.4978, and SED *p* = 0.5539).

### 3.3. Different Pattern of Th Immune Response in Upper Airway in the RUN and SED Groups

**Figure 5:**
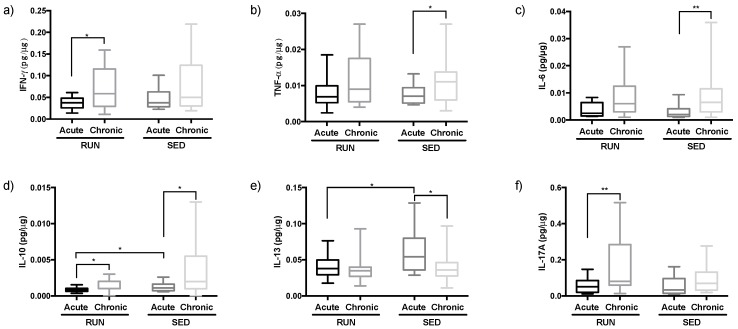
Comparison of the cytokine levels of interferon (IFN)-γ (**a**), tumor necrosis factor (TNF)-α (**b**), interleukin (IL)-6 (**c**), IL-10 (**d**), IL-13 (**e**), and IL-17A (**f**) between RUN and SED groups and between time points (after acute exposure (AE) and after chronic exposure (CE)). All data were analyzed using the Welch two-tailed unpaired test. Values are presented in median and with respective quartile. Level of significance was established at 5% (*p* < 0.05).

As observed in Figure 5, the levels of IL-10 (Figure 5d, *p* = 0.03) and IL-13 (Figure 5e, *p* = 0.02) were lower after AE in the RUN group than in SED group. No differences were found after CE between the groups. In the analysis between time points (AE × CE), higher levels of IFN-γ (Figure 5a, *p* = 0.02), IL-10 (Figure 5d, *p* = 0.01), and IL-17A (Figure 5f, *p* = 0.005) were found after CE than after AE in the RUN group. Regarding the SED group, higher levels of TNF-α (Figure 5b, *p* = 0.04), IL-6 (Figure 5c, *p* = 0.004), and IL-10 (Figure 5d, *p* = 0.01) and also lower IL-13 levels (Figure 5e, *p* = 0.02) were observer after CE compared to after AE values.

Table 3 shows the analysis of the ratios between time points. In the RUN group, only the ratio between TNF-α and IL-13 (reflecting Th1/Th2 immune response) showed a significant difference after CE compared to AE values. Although in RUN the levels of TNF-α and IL-13 only tended to differ between CE and AE (Figure 5b,d), the ratio of these cytokines made the increase of Th1 immune response after CE also visible in RUN. In addition, in RUN, the ratio between IL-13 and IL-17A (reflecting Th2/Th17 immune response) showed a significant decrease after CE compared to the AE values, mainly due to the significantly increased IL-17A levels after CE. So, in runners, chronic exposure to PM changes the pattern of cytokines towards a more Th1/Th17 driven immune response. Similar to the RUN group, the ratio between IL-13 and IL-17A (reflecting Th2/Th17 immune response) significantly increases after CE compared to AE. Instead, it is mainly due to decreased IL-13 levels after CE, rather than to increased IL-17A levels, however, the net ratio result is comparable.

In contrast to the RUN group, in the SED group, the ratio between TNF-α and IL-6, and TNF-α and IL-10 (reflecting Th1/Th2 immune response) showed significant differences when comparing AE with CE. Although the levels of TNF-α, IL-6, and IL-10 all increased after CE compared to after AE, the extent of the increase for IL-6 and IL-10 was higher than for TNF-α, which in SED favors a Th2 immune response. Corroborating this result, the higher levels of IL-10 after CE when compared to AE had its impact on the ratio between IL-10 and IFN-γ, also favoring a Th2/IL-10-dominated immune response.

In order to evaluate the pattern of Th immune response in the RUN and SED groups, after AE and CE, the ratio between the Th1 cytokines (IFN-γ, TNF-α), Th2 cytokines (IL-6, IL-10, and IL-13) and also Th17 cytokine (IL-17A) were performed.

**Figure 6:**
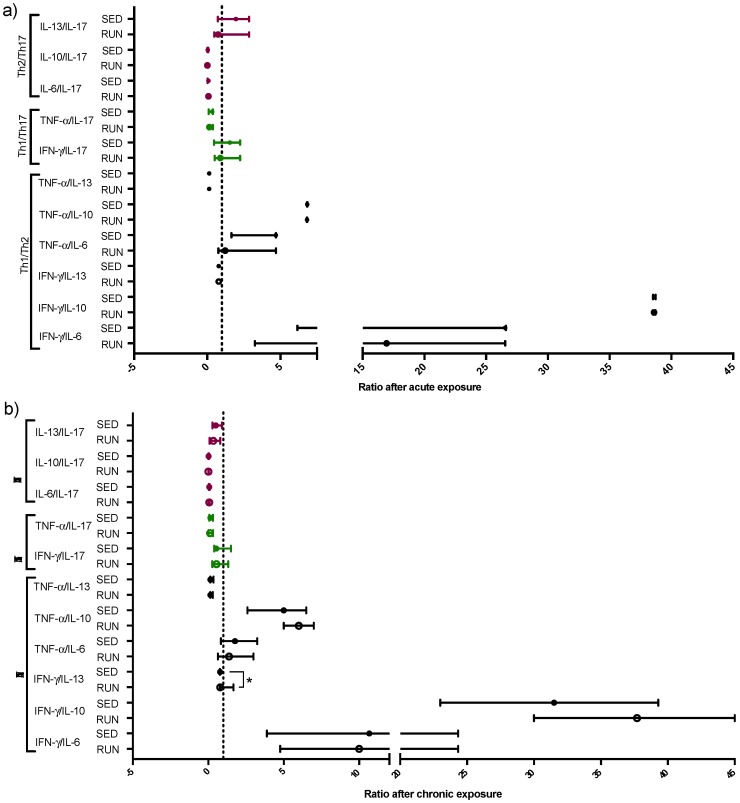
Comparison of Th1/Th2, Th1/Th17, and Th2/Th17 immune response between RUN and SED groups after acute (**a**) and chronic (**b**) exposure. The dotted line represents the value 1. All data were analyzed using the Welch two-tailed unpaired test. Values are presented in median and with respective quartile. Level of significance was established at 5% (*p* < 0.05).

As observed in Figure 6, the ratio between IFN-γ and IL-13 (Th1/Th2 immune response) the RUN group showed predominant Th1 response after CE compared to the SED group (Figure 6b, *p* = 0.02). No significant differences were found between the other ratios.

### 3.4. Salivary Immunological Markers are Increased in Runners

**Figure 7:**
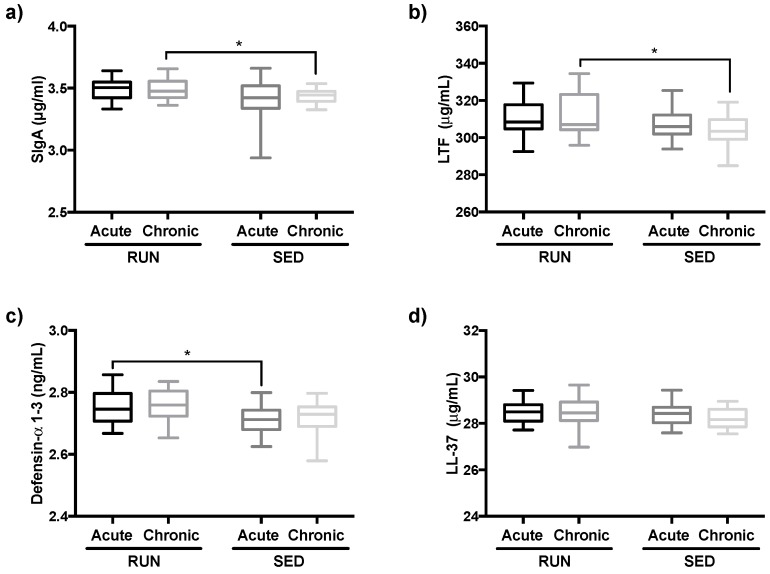
Comparison of secretory immunoglobulin A (SIgA (**a**) and antibacterial peptides lactoferrin (LTF) (**b**), defensin-α 1-3 (**c**), LL-37 (**d**)) levels in saliva of RUN and SED groups after AE and CE. All data were analyzed using the Welch two-tailed unpaired test. Values are presented in median and with respective quartile. Level of significance was established at 5% (*p* < 0.05).

As shown in Figure 7, the comparison between the groups showed higher levels of defensin-α 1–3 in the RUN group after AE than in the SED group (Figure 7c, *p* = 0.03). In addition, higher levels of SIgA and LTF were found in the RUN group after CE as compared to the SED group (Figure 7a, *p* = 0.03, and Figure 7b, *p* = 0.02, respectively). No differences were observed in salivary LL-37 levels (Figure 7c). In the comparison between the time points, no significant differences were found between the groups.

## 4. Discussion

Our results showed that runners, compared to sedentary subjects, can profit from increased immunological protecting mechanisms on different levels. First, we observed the fact that the salivary levels of antibacterial peptides and SIgA were higher both after acute (salivary defensins) and chronic (SIgA, LTF) exposure to particulate matter (and/or intense exercise) in RUN compared to SED. In agreement with results from other authors, some agents, such as SIgA and antibacterial peptides, are involved in the improvement of airway mucosal immunity by directly inhibiting the actions of pathogens in this environment. Although SIgA is considered as the main agent in the mucosa, most recently, the importance of antibacterial peptides in the protection of airway mucosa has been recognized [41,42]. Higher levels of these immunological agents can be found in physically active people [43,44,45] leading to greater protection, which decreases the incidence of upper respiratory tract infection in this population [42].

In terms of the effect of particulate matter exposure on antibacterial peptides levels, it has been reported that particulate matter can promote a reduction of airways antibacterial peptides, thereby enabling pathogens to adhere to the airway epithelial cells, and consequentially increase airway infection [46]. Corroborating this information, it was found that acute exposure leads to a reduction of defensin [47]. In a different way, our results showed that after AE the salivary defensin levels were higher in runners than in sedentary people, demonstrating a positive effect of exercise training in the maintenance of mucosal immunity. According to other authors, pollutant exposure elicits a mucosal immune response with an increase of antibacterial peptides, such as lactoferrin [48,49], whereas the influence of pollutants such as PM_2.5_ and PM_10_ on salivary levels of SIgA have shown conflicting results, since some studies showed no change [50], or reduction [51,52,53], or increase in salivary levels after exposure [54]. Based on our data, we can suggest that the hyperventilation that occurs during the outdoor endurance practice, which induces the accumulation of particles in the airways [24], can stimulate the increase of lactoferrin and SIgA, that was found after CE. So, the association of chronic particulate matter exposure and the particle accumulation in airways was able to elicit an immunological response, improving the mucosal airway protection, albeit only in runners.

Secondary, in runners the fraction of exhaled NO (FeNO) is always significantly lower in RUN when compared to SED, whether after acute or chronic exposure to PM. FeNO is a remarkable biomarker presenting increased levels in association with pulmonary inflammation, mainly in situations with an evident presence of eosinophils infiltrating in the airways. According to the American Thoracic Society [37], FeNO levels higher than 25 ppb are clinically significant cut-off points for eosinophilic inflammation [37,55]. In the literature, it is widely accepted that elevations in the concentration and also deposition of air pollutant particles in the airways are closely associated with lung inflammation [56]. In relation to physical exercise, this fact is corroborated by data reported by Thornadtsson et al. [57], in which amateur runners showed increased FeNO levels after a marathon race as compared to sedentary people, since during an endurance exercise, such as a marathon race the runners presented hyperventilation that leads to higher deposition of air pollutants in the airways [24]. In a different way, our results demonstrated that both absolute FeNO levels as well as the proportion of volunteers presenting levels above 25 ppb, were lower in the RUN group than in the SED group whether after AE or after CE at rest. Although the RUN group presented with lower FeNO levels, these reduced values were associated with significantly lower plasma CC16 levels in this group after AE (*p* = 0.06) nor after CE (*p* = 0.08) when compared to the SED group. Plasma analysis of CC16 protein, in general, can be seen as a marker of injury in the pulmonary epithelial barrier integrity [58,59]. We can conclude that, in the SED group, the higher FeNO levels indicate that pulmonary inflammation might be particularly present in this group, whereas potential epithelial damage, which is known to be induced by exposure to PM [60] seems similar in both groups. It is also worthy to mention that the analysis of FeNO levels comparing the data found after AE and after CE in runners group showed higher FeNO levels after AE. This finding corroborates the literature, since that, in a general way, increases in the FeNO levels occurs acutely in response to air pollutants exposure challenge [57].

Thirdly, looking for the nasal cytokine profile associated with PM exposure, runners showed stable Th2 related cytokine expression after acute PM exposure, whereas in the sedentary group acute exposure to PM is specifically associated with increased nasal fluid IL-13 levels. Elevation in nasal lavage fluid IL-13 levels and an increase in eosinophilic inflammation were similarly found in mice exposed to PM_2.5_ [61] and to PM_10_ [62]. The production of nitric oxide in the airway epithelium can be induced by IL-13, which is confirmed after the blockage of IL-13 with an antagonist, resulting in reduced FeNO levels [63,64]. Although IL-13 in our subjects was measured nasally, due to the ‘united airways’ theory, it is tempting to speculate about its presence in the lower airways, which might be increased in parallel. This would explain why FeNO levels are also specifically increased in SED compared to RUN.

Moreover, after chronic exposure, we observed a significant reduction of IL-13 levels in the SED group, which could be related to the tendency of decreased FeNO levels in this group after chronic exposure, whereas in the RUN group FeNO levels again remained unchanged. However, the reduction in IL-13 levels in SED could also be influenced by the increased levels of IL-6 and IL-10 in that group, as these cytokines are all known to be involved in the Th2 immune response and interact with each other [65]. On the other hand, IL-13 is reported to stimulate alveolar macrophages to polarize into the M2 profile, by which a significant release of IL-10 in the respiratory tract can be induced [66]. In addition, particulate matter exposure by itself can also induce macrophage M2 polarization [66]. So we might suppose that M2 polarization due to acute PM exposure could also explain the elevation of IL-10 levels in both SED and RUN groups, but that the high levels of IL-13 in SED later on (upon chronic exposure) induced a higher IL-10 induction when compared to RUN due to additional M2 polarization induction. Although we here considered IL-10 to be a Th2 cytokine, it is widely accepted that IL-10 might also have anti-inflammatory properties and can act as a factor to protect the airways against mucosal inflammation [67]. Moreover, besides M2, epithelial cells were also reported to release IL-10, which can induce T-regulatory (T-reg) cell differentiation, involved in the induction of a non-inflammatory microenvironment that favors homeostasis [67,68]. We also demonstrated earlier that IL-10 in the airways can inhibit the manifestation of airway symptoms after running a marathon, a situation in which of course also increased exposure to air pollutants occurs [67]. Therefore, the higher IL-10 levels in both groups after chronic PM exposure compared to acute exposure can furthermore protect the airway environment against the harmful effects of chronic particulate matter exposure. Corroborating this anti-inflammatory property, the higher IL-10 levels observed in SED group after CE are responsible for the decrease in the ratio between IFN-γ or TNF-α and IL-10, however it is not sufficient to reduce the Th2 (IL-13) immune response in this volunteer’s group.

In relation to IL-6, it was demonstrated that particulate matter exposure could lead to a significant increase of this cytokine in the bronchoalveolar lavage of mice [69], airways epithelial cells [70], and alveolar macrophages [71]. So, similar chronic exposure to particulate matter could lead to the elevation of IL-6 levels in the respiratory tract, as found in the SED group. In addition, higher IL-6 levels impact a decreased ratio between TNF-α and this cytokine, while maintaining the Th2 (IL-13) immune response, similar to what was observed for IL-10 [67]. So, this suggests that the maintenance of the Th2 immune response and especially the higher levels of IL-6 can impair the respiratory tract of sedentary people during chronic particulate matter exposure and counteract the potentially beneficial effects of IL-10. It is worth to report that higher levels of IL-6 are presented in different airway diseases in which a Th2 immune response is associated, such as allergic [72], chronic obstructive pulmonary disease (COPD) [73], and asthma [74]. Regarding exercise, Vaisberg et al. showed that increased IL-6 levels were associated with inflammation in the respiratory tract and also with manifestation of symptoms in upper airways in symptomatic marathon runners, whereas the asymptomatic runners showed good control of inflammation. Although we observed a tendency towards higher nasal IL-6 expression in runners after chronic exposure to PM, we, unfortunately, have no data on symptoms in these runners, which could eventually explain the increase in IL-6 in some but not all runners. Another point that deserves to be highlighted is that IL-6 is involved in the differentiation of T cells towards Th17 cells [74]. Although IL-6 and IL-17A levels both in runners and in sedentary subjects show parallel increase after chronic exposure, they do not completely correlate with each other. Indeed, IL-17A levels only increased significantly in runners. IL-17 increased in the airways after intense exercise has been shown in other reports [75]. IL-17A levels can furthermore be induced by chronic PM_2.5_ exposure [76]. Higher IL-17A levels released by CD4+ Th17 lymphocytes can stimulate airway epithelial cells to secrete several factors involved, not only in the physiological regulation [77], but also in the airway protection [78] and it has been supposed that the Th17 immune response can protect the airways e.g., against microorganisms in which the Th1 and Th2 immune responses are not sufficient to guarantee the protection by themselves [78]. Therefore, the elevation of IL-17A levels can also reflect the maintenance and protection of the airway mucosa against other factors, such as chronic particulate matter exposure in runners. Nevertheless, the ratio between IL-13 and IL-17A in runners as well as in sedentary subjects was lower after CE compared to AE showing that a Th17 immune response is present in both groups. Interestingly, the pattern of Th17 immune response, though observed in both groups, was due to different reasons. It has been demonstrated that in the pathogenesis, inflammation, and severity of airway disease, the association between Th2 and Th17 immune responses is involved [74,78,79,80,81]. This finding can putatively reinforce our suggestion that the chronic particulate matter exposure is harmful. However, in runners more mechanisms might be present to balance these potential harmful factors when compared to sedentary subjects.

Beyond the increase of IL-17A levels, the RUN group also showed higher levels of nasal IFN-γ and IL-10 after chronic particulate matter exposure, similar to what was already described by other authors and supposed to be macrophage-dependent [76]. Regarding the elevated IFN-γ (a classical cytokine presenting in Th1 immune response) levels, it is widely accepted that this cytokine is associated with protection against infection induced by viruses and other microorganisms, in a variety of cells including airway epithelial cells [82]. Based on these data and our results, the regular practice of exercise can enhance airway protection, since the ratio between IFN-γ and IL-13 was higher in the RUN group than in the SED group after CE, showing that a Th1 immune profile was elicited, in contrast to the Th2 immune response presenting in the SED group.

Taken together, all the results corroborate our hypothesis that the practice of outdoor aerobic exercises was able to modulate the airways’ immune/inflammatory responses in a different way than found in sedentary people.

## 5. Conclusions

The novelty of this study was that the long-standing regular practice of outdoor endurance exercise might improve the immunological and inflammatory status in the airways after both acute and chronic particulate matter exposure differently from that observed in the sedentary lifestyle of people in the same community.

## Figures and Tables

**Table 1:** Physical characteristics (gender and its ratio, age, weight, height, body mass index (BMI), total body fat, and maximal oxygen uptake ($\dot{V}$O_2max_)) of the volunteers allocated in the groups RUN and SED. All data were analyzed using Student’s t-test and are presented in mean and standard deviation (SD). Level of significance was established at 5% (*p* < 0.05).

	Runners	Sedentary	*p* Value
**Number (n)**	20	20	
**Gender**			
**Women (n)**	4	8	
**Men (n)**	16	12	
**Ratio (W/M)**	1/4	1/1.5	0.1675
**Age (years)**	37.4 ± 8.99	31.8 ± 10.27	0.0746
**Weight (kg)**	73.9 ± 16.21	77.6 ± 19.65	0.5267
**Height (cm)**	172 ± 9.72	172.2 ± 10.02	0.9527
**BMI**	24.7 ± 3.81	26.0 ± 5.65	0.6964
**Total body fat (%)**	24.9 ± 6.69	30.5 ± 7.17	0.0187
**$\dot{V}$O_2max_ (mL/kg/min)**	41.8 ± 6.54	29.3 ± 5.02	<0.0001

Note: Body mass index (BMI); Maximal oxygen uptake ($\dot{V}$O_2max_).

**Table 2:** Average levels of pollutants (mean ± standard deviation) at different time intervals and the ratio of the concentration of pollutants in the sampling week to the concentration of pollutants in previous weeks. All data were analyzed using the Welch two-tailed unpaired test. Level of significance was established at 5% (*p* < 0.05).

Weeks for Measuring Pollutants	PM_2.5_	PM_10_	O_3_
Five weeks prior to first sampling day	17.72 ± 9.6	27.07 ± 13.76	19.75 ± 8.24
First sampling week	32.24 ± 12.28 *	50.18 ± 15.56 ^#^	22.67 ± 5.2
Four weeks prior to the sampling week	14.71 ± 5.52	22.29 ± 6.9	19.14 ± 8.68
Ratio between sampling week and the previous 4 weeks	2.19	2.25	1.18
Ten weeks between sampling days	22.49 ± 8.90	37.39 ± 15.23	29.18 ± 8.77
Nine weeks prior to second sampling day	21.66 ± 8.52	31.29 ± 8.17	35.96 ± 14.22
Second sampling week	20.8 ± 12.6	34.47 ± 18.55	37.24 ± 3.7
Ratio between sampling week and the previous 9 weeks	0.96	1.1	1.03

Note: *,^#^ Difference between first sampling week and four weeks previous sampling week.

**Table 3:** Comparison of average levels of cytokines ratio between Th1/Th2, Th1/Th17, and Th2/Th17 immune response at different time points in both the RUN and SED group. All data were analyzed using the Welch two-tailed unpaired test. Level of significance was established at 5% (*p* < 0.05).

			Runners		Sedentary	
			Mean ± SD	*p*	Mean ± SD	*p*
Th1/Th2	IFN-γ/IL-6	Acute	15.81 ± 2.90	0.702	17.25 ± 2.61	0.493
		Chronic	12.48 ± 2.18		16.27 ± 3.42	
	IFN-γ/IL-10	Acute	38.61 ± 0.04	0.791	38.61 ± 0.03	0.016
		Chronic	43.35 ± 4.20		32.51 ± 3.04	
	IFN-γ/IL-13	Acute	0.78 ± 0.00	0.091	0.78 ± 0.00	0.558
		Chronic	1.21 ± 0.20		0.77 ± 0.07	
	TNF-α/IL-6	Acute	2.88 ± 0.65	0.393	3.20 ± 0.39	0.035
		Chronic	1.89 ± 0.38		2.15 ± 0.35	
	TNF-α/IL-10	Acute	6.81 ± 0.00	0.052	6.83 ± 0.00	<0.0001
		Chronic	6.18 ± 0.50		4.36 ± 0.53	
	TNF-α/IL-13	Acute	0.13 ± 0.00	0.023	0.13 ± 0.00	0.397
		Chronic	0.21 ± 0.02		0.20 ± 0.03	
Th1/Th17	IFN-γ/IL-17	Acute	1.23 ± 0.18	0.126	1.40 ± 0.22	0.101
		Chronic	0.88 ± 0.18		0.95 ± 0.17	
	TNF-α/IL-17	Acute	0.22 ± 0.03	0.149	0.31 ± 0.05	0.179
		Chronic	0.15 ± 0.032		0.17 ± 0.02	
Th2/Th17	IL-6/IL-17	Acute	0.08 ± 0.013	0.423	0.06 ± 0.00	0.922
		Chronic	0.06 ± 0.00		0.06 ± 0.01	
	IL-10/IL-17	Acute	0.02 ± 0.00	0.392	0.03 ± 0.00	0.134
		Chronic	0.02 ± 0.00		0.02 ± 0.00	
	IL-13/IL-17	Acute	1.34 ± 0.25	0.015	1.80 ± 0.25	0.001
		Chronic	0.53 ± 0.14		0.54 ± 0.10	

Note: T helper (Th); interferon (IFN); interleukin (IL); tumor necrosis factor (TNF). Mann-Whitney comparison, *p* < 0.05.
